# Forsythoside A Alleviates Imiquimod-Induced Psoriasis-like Dermatitis in Mice by Regulating Th17 Cells and IL-17A Expression

**DOI:** 10.3390/jpm12010062

**Published:** 2022-01-06

**Authors:** Hsuan Lin, Chia-Ling Li, Ling-Jung Yen, Ling-Ying Lu, Hung-Sen Huang, En-Chih Liao, Sheng-Jie Yu

**Affiliations:** 1 Department of Traditional Chinese Medicine, Kaohsiung Veterans General Hospital, Zuoying, Kaohsiung 813, Taiwan; hlin@vghks.gov.tw; 2 Children’s Medical Center, Taichung Veterans General Hospital, Xitun, Taichung 407, Taiwan; lingboxer@gmail.com; 3 Division of Allergy, Immunology, and Rheumatology, Department of Medicine, Kaohsiung Veterans General Hospital, Zuoying, Kaohsiung 813, Taiwan; ljung@vghks.gov.tw (L.-J.Y.); lylu@vghks.gov.tw (L.-Y.L.); 4 Department of Medical Education and Research, Kaohsiung Veterans General Hospital, Zuoying, Kaohsiung 813, Taiwan; daniel0822@gmail.com; 5 Department of Medicine, MacKay Medical College, Sanzhi, New Taipei City 252, Taiwan; enchih@mmc.edu.tw; 6 Institute of Biomedical Sciences, MacKay Medical College, Sanzhi, New Taipei City 252, Taiwan

**Keywords:** forsythoside A, *Forsythia suspensa*, psoriasis, IL-17A, Th17 cells

## Abstract

Psoriasis is a recurrent inflammatory skin disease characterized by redness and scaly skin lesions with itchy or painful sensations. Forsythoside A, one of the main active compounds isolated from the fruit of *Forsythia suspensa*, has been widely applied to treat inflammatory diseases in the clinical use of traditional oriental medicine. However, the effect of forsythoside A on psoriasis remains unclear. This study aimed to explore the therapeutic effects and immune regulation of forsythoside A on psoriasis. C57BL/6 mice were divided into six groups and treated with imiquimod cream on their shaved back skin to induce psoriasis-like dermatitis. Different doses of forsythoside A (5 mg/kg, 10 mg/kg, or 20 mg/kg) were administered to the respective treatment groups. Skin redness, scaling, and ear thickness were measured; keratinocyte proliferation and inflammatory cytokine expression were detected by hematoxylin–eosin and immunohistochemical staining. Th17 cells in the inguinal lymph nodes were detected by flow cytometric analysis. IL-17A levels were measured using ELISA. The results showed that forsythoside A relieved psoriatic skin symptoms such as skin redness, thickness, scaling, and reduced epidermal thickening. The expression of IL-6, IL-17, and Ki-67 was downregulated in the forsythoside-A-treated groups. Th17 cell expression in inguinal lymph nodes and IL-17A secretion was suppressed by forsythoside A. In conclusion, forsythoside A was found to alleviate imiquimod-induced psoriasis-like dermatitis in mice by suppressing Th17 development and IL-17A secretion. These findings demonstrate the feasibility of forsythoside A in treating human psoriasis.

## 1. Introduction

Psoriasis is a common chronic, noninfectious, and recurrent inflammatory skin disease [1]. A systematic review reported that the worldwide prevalence of psoriasis ranged from 0.5% to 11% in adults and 0% to 1.4% in children, with no clear gender bias [1,2]. The incidence rates were found to be bimodal, ranging from 30 to 39 years and 50 to 69 years [1]. Psoriasis can present with variable morphology and severity, characterized by circular, red papules, or plaques with a silvery-white dry scale, accompanied with painful or itchy sensations [3]. The lesions are typically located symmetrically on the scalp, extremities, and trunk, sometimes in the body folds or at the site of trauma or injury [3]. Psoriasis vulgaris, also known as plaque-type psoriasis, is the most common subtype. Psoriasis affects the skin as well as other organ systems, leading to various diseases such as coronary artery disease, hypertension, diabetes mellitus, inflammatory bowel disease, chronic kidney disease, and psoriatic arthritis [4,5]. In addition to the physical dimensions, mood disorders have been found to be more prevalent in patients with psoriasis than in the general population, leading to anxiety and suicidal ideation [5]. Overall, psoriasis can tremendously diminish the patients’ quality of life.

Psoriasis is characterized by continuous inflammation that results in uncontrolled keratinocyte proliferation and dysfunctional differentiation. The histology of psoriatic plaques shows epidermal hyperplasia with inflammatory infiltrates consisting of T cells, dermal dendritic cells, neutrophils, and macrophages [6]. Early experiments have found that the inflammatory infiltrates in psoriatic skin lesions largely comprise CD4+ and CD8+ T cells [6], indicating that psoriasis is a T-cell-mediated disease. A subsequent study indicated that the IL-23/Th17 signaling pathway plays a key role in inducing psoriasis [7]. Driven by IL-23 in prepsoriatic skin, activated Th17 cells produce large amounts of IL-17, including IL-17A and IL-17F, as well as other cytokines such as IL-26, IL-29, and TNF, thus inducing a feedforward inflammatory response in keratinocytes, and finally, the development of mature psoriatic skin lesions [6,7].

In the context of traditional therapy choices, mild or limited psoriatic lesions are usually treated with topical steroids, emollients, and tar. Several new types of drug carriers such as liposomes, gels, and foams, have also been developed to promote the effects of topical therapy [8]. In patients with moderate to severe psoriasis or symptoms that cannot be treated ideally, systemic therapy or phototherapy might be considered. However, poor compliance for some kinds of systemic therapy has been demonstrated due to the underlying liver and kidney toxicity, and the concern of cancer risk with phototherapy, which cannot be ignored [9]. Biological agents such as TNF-α and IL-17/IL-23 inhibitors confer a heavy economic burden to patients and their families due to their high costs, despite their safety and tolerance [9].

In the clinical practice of traditional oriental medicine, the fruit of *Forsythia suspensa* has been widely applied to treat inflammatory diseases, because it possesses antipyretic effects of heat-toxin clearing and swelling-lump elimination. In modern pharmacology, previous studies indicated that it exerts anti-inflammatory effects in a murine model of atopic dermatitis by inhibiting chemokine production in TNF-α/IFN-γ-activated human keratinocytes [10]. Forsythoside A, one of the main active compounds isolated from the fruit of *Forsythia suspensa*, is also reported to have a significant antiviral effect because it inhibits influenza A virus replication by downregulating the mRNA expression of TLR7 signaling pathway components [11]. However, the effect of forsythoside A on psoriasis remains unclear. Therefore, we aimed to explore the therapeutic effect of forsythoside A in treating psoriasis by examining the modulation of the immune response and pro-inflammatory cytokine expression in vitro and in vivo.

## 2. Materials and Methods

### 2.1. IMQ-Induced Psoriasis-like Dermatitis Model

Eight-week-old C57BL/6 mice were purchased from the National Laboratory Animal Center (Taipei, Taiwan). The mice were housed at Kaohsiung Veterans General Hospital (KSVGH), Kaohsiung, Taiwan, in a specific pathogen-free facility, at a constant temperature of 23 ± 1 °C, relative humidity of 50–60%, and a 12:12 h light:dark cycle; they were provided with free access to chow and water. The Institutional Animal Care and Use Committee of KSVGH approved all the animal experimental protocols (2021-A039).

In the early treatment model, mice were randomly separated into six groups (*n* = 5 in each group): control group, vehicle control group, forsythoside A (CAS No. 79916-77-1, purchased from Chengdu Biopurify Phytochemicals Ltd., Chengdu, China) groups (5 mg/kg, 10 mg/kg, 20 mg/kg), and a dexamethasone group (1 mg/kg). For the murine model of IMQ-induced psoriasis, 62.5 mg of IMQ cream (Aldara, 3M Pharmaceuticals, Saint Paul, MN, USA) was topically applied on the shaved backs and left ears of mice. Except for the control group, all mice were topically treated with 62.5 mg IMQ for 5 days. In the vehicle control group, mice were intraperitoneally injected with 3% DMSO in 200 μL normal saline daily. In the forsythoside A group, mice were intraperitoneally injected with 5 mg/kg, 10 mg/kg, or 20 mg/kg forsythoside A in 200 μL normal saline daily. In the dexamethasone group, mice were intraperitoneally injected with 1 mg/kg dexamethasone in 200 μL normal saline daily. Skin redness, scaling (scale from 0 to 4; 0, none; 1, slight; 2, moderate; 3, severe; 4, very severe), ear thickness was measured, and the mouse back skin was photographed daily. On day 6, the mice were sacrificed, and the skin and inguinal lymph nodes were collected for further experiments.

In the treatment model, mice were also randomly separated into six groups (*n* = 5 in each group): control group, vehicle control group, forsythoside A groups (5 mg/kg, 10 mg/kg, 20 mg/kg), and a dexamethasone group (1 mg/kg). Except for the control group, 62.5 mg of IMQ cream was topically applied on the shaved backs and left ears of mice for 7 consecutive days. In the vehicle control group, mice were intraperitoneally injected with 3% DMSO in 200 μL normal saline from day 3 to day 7. In the forsythoside A group, mice were intraperitoneally injected with 5 mg/kg, 10 mg/kg, or 20 mg/kg forsythoside A in 200 μL normal saline from day 3 to day 7. In the dexamethasone group, mice were intraperitoneally injected with 1 mg/kg dexamethasone in 200 μL normal saline from day 3 to day 7. Skin redness, scaling (scale from 0 to 4; 0, none; 1, slight; 2, moderate; 3, severe; 4, very severe), and ear thickness was also measured, and the mouse back skin was also photographed daily. On day 8, the mice were sacrificed.

### 2.2. Flow Cytometry and Intracellular Staining (ICS)

Immune cells isolated from inguinal lymph nodes were activated with 50 ng/mL phorbol 12-myristate 13-acetate (PMA, Sigma-Aldrich, St. Louis, MO, USA) and 500 ng/mL ionomycin (Sigma-Aldrich, USA) and monensin (BD Biosciences, San Jose, CA, USA) for 5 h at 37 °C in a humidified atmosphere containing 5% CO_2_. The cells were harvested and washed prior to staining with anti-CD4 PerCP (BioLegend, San Diego, CA, USA) and then incubated with fix/permeabilize solution (BD Cytofix/CytopermTM, San Jose, CA, USA) for 30 min at 4 °C, followed by intracellular cytokine staining. Cells were stained with anti-IL-17 PE and anti-IFN-γ FITC (BioLegend, San Diego, CA, USA). After staining, samples were detected using a flow cytometer (FACSCalibur, BD, San Jose, CA, USA) and the data were analyzed using FlowJo software (FlowJo, LLC., Ashland, OR, USA).

### 2.3. Cell Viability and Flow Cytometry Analysis of Apoptosis

Lymphocytes were harvested from C57BL/6 mice and activated with plate-bound anti-CD3 (5 μg/mL; BioLegend, San Diego, CA, USA) and soluble anti-CD28 (1 μg/mL; BioLegend, San Diego, CA, USA), anti-IFN-γ (10 μg/mL; BioLegend, San Diego, CA, USA), anti-IL-4 (10 μg/mL; BioLegend, San Diego, CA, USA), IL-1β (20 ng/mL; Peprotech, East Windsor, NJ, USA), IL-6 (20 ng/mL; Peprotech, East Windsor, NJ, USA), IL-23 (20 ng/mL; BioLegend, San Diego, CA, USA) and TGF-β1 (1.25 ng/mL; BioLegend, San Diego, CA, USA). Lymphocytes were then treated with different doses of forsythoside A, and dexamethasone was used as the control, for 4 days in a 96-well plate.

After treatment with the forsythoside A, the cell viability of lymphocytes was measured with a CCK-8 commercial kit (Sigma-Aldrich, St. Louis, MO, USA), following the manufacturer’s protocol. Briefly, cells were washed with PBS followed by 100 μL/well CCK-8 working reagent added into the culture plate. Cells were incubated in the incubator at 37 °C, 5% CO_2_ for 2 h. The absorbance at 450 nm was measured using a plate reader (SUNRISE; Tecan Group Ltd., Männedorf, Switzerland). Cell apoptosis was detected by propidium iodide (PI) stain. Briefly, after incubation with different doses of forsythoside A, lymphocytes were stained with PI (3 μM) (Sigma-Aldrich, St. Louis, MO, USA) for 30 min at room temperature, and the cell samples were analyzed by flow cytometer (FACSCalibur, BD, San Jose, CA, USA).

### 2.4. Histological and Immunohistochemical Stain

The dorsal skin of each mouse was cut and fixed in formalin solution, embedded in paraffin, cut into 5 μm sections, and stained with hematoxylin and eosin (H&E). For immunohistochemical staining, after deparaffinization, antigen retrieval, and blocking, 5 μm sample sections were incubated overnight with primary antibodies against IL-6, IL-17, and Ki-67 (Abcam; Burlingame, CA, USA). The sections were then washed and incubated with an antibody enhancer (Cell Marque; Rocklin, CA, USA) for 10 min at room temperature. After washing, the sections were incubated with horseradish peroxidase (HRP)-conjugated antibodies (Cell Marque; USA) for 10 min at room temperature. The sections were then washed and developed with 3,3′-Diaminobenzidine (DAB, Cell Marque; Rocklin, CA, USA) for 2 min at room temperature and counterstained with hematoxylin. The sample section was visualized and imaged using a digital camera (Axiocam 105; Jena, Germany) mounted on a phase contrast microscope (Zeiss, Jena, Germany) with Zen 2.3 software (Zeiss, Jena, Germany). The mean optical density was analyzed with ImageJ software (version 1.50i, National Institutes of Health, Bethesda, MD, USA).

### 2.5. In Vitro Lymphocyte Culture and ELISA

Lymphocytes were harvested from C57BL/6 mice and activated with plate-bound anti-CD3 (10 μg/mL; BioLegend, San Diego, CA, USA) and soluble anti-CD28 (1 μg/mL; BioLegend, San Diego, CA, USA). Lymphocytes were then treated with different doses of forsythoside A, and dexamethasone was used as the control, for 24 h in a 24-well plate. After incubation with conditioned medium, the supernatant harvested from the lymphocyte culture was centrifuged to remove the floating cells. IL-17A production was measured using a sandwich ELISA method, according to the manufacturer’s protocol (BioLegend, USA). After incubating the conjugate with 3,3′,5,5′-tetramethylbenzidine (TMB, BioLegend, USA), the absorbance at 450 nm was measured using a plate reader (SUNRISE; Tecan Group Ltd., Männedorf, Switzerland).

### 2.6. Statistics

Data analysis was performed using GraphPad Prism 6 (GraphPad, San Diego, CA, USA). One-way ANOVA or two-way ANOVA with LSD multiple comparison post-tests was used to compare the variance between groups. Statistical significance was set at *p* < 0.05. The results are presented as the mean ± SEM.

## 3. Results

### 3.1. Forsythoside A Relieves the Symptoms of Imiquimod-Induced Psoriasis-like Dermatitis

**Figure 1:**
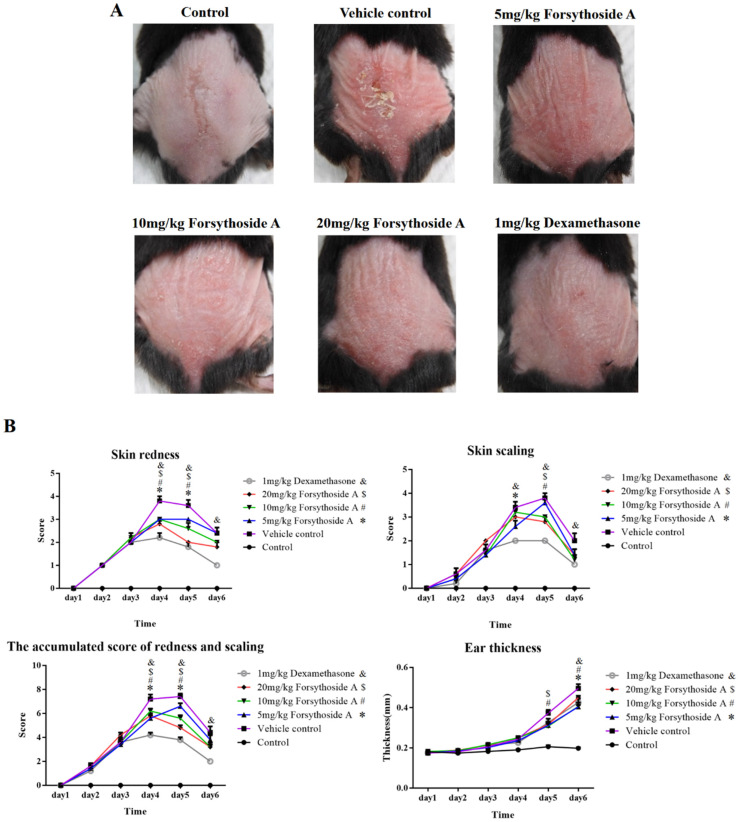

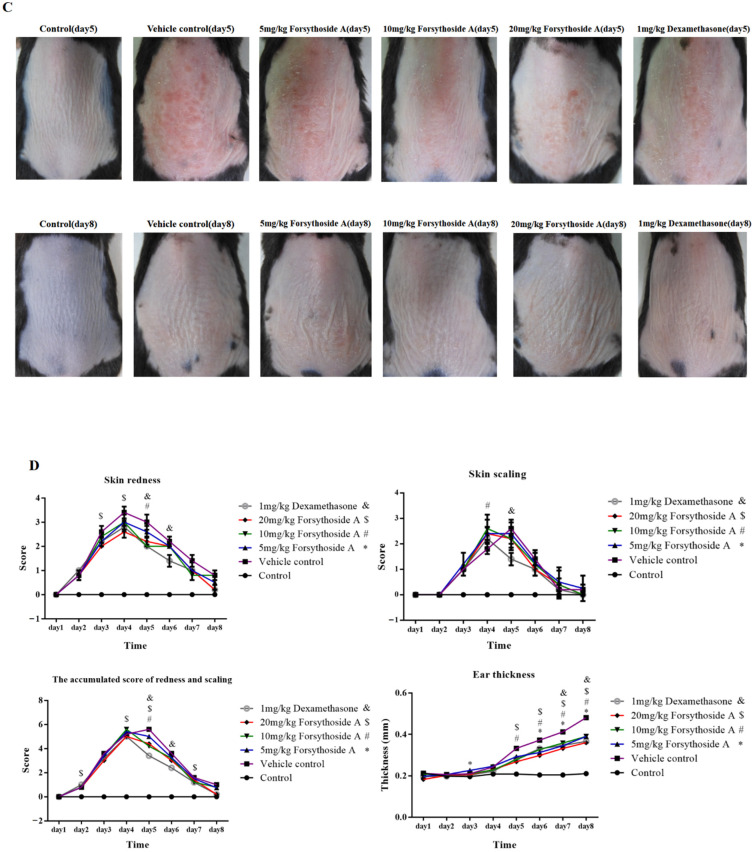
Forsythoside A improved the morphological features in a psoriasis-like murine model. In the early treatment model, except for the control group, C57BL/6 mice were treated daily with IMQ cream on their shaved back skin for 5 consecutive days to induce psoriasis-like dermatitis, and treated with forsythoside A at 5, 10, or 20 mg/kg simultaneously; the mice were then sacrificed on day 6. In the treatment model, IMQ cream was applied to C57BL/6 mice for 7 consecutive days, and then treated with forsythoside A at 5, 10, or 20 mg/kg from day 3 to day 7. Mice in the vehicle control group were treated with 3% DMSO in 200 μL normal saline. Mice treated with 1 mg/kg of dexamethasone were used as the control group. (**A**) Phenotypical presentation of the back skin in the early treatment model at day 6. (**B**) In the early treatment model, redness and scaling of the back skin were scored daily on a scale from 0 to 4, and ear thicknesses were measured using a scale (mm). The accumulated scores of redness and scaling on the back skin are presented. (**C**) Phenotypical presentations of the back skin in the treatment model at day 5 and day 8. (**D**) In the treatment model, redness and scaling of the back skin were scored daily on a scale from 0 to 4, and ear thickness were measured using a scale (mm). The accumulated scores of redness and scaling on the back skin are presented. Data were presented as the mean ± SEM (*n* = 5 in each group). *, #, $, & *p* < 0.05 vs. vehicle control group. * 5 mg/kg forsythoside A; # 10 mg/kg forsythoside A; $ 20 mg/kg forsythoside A; & 1 mg/kg dexamethasone.

The therapeutic effects of forsythoside A were investigated in an imiquimod-induced murine model of psoriasis. Topical use of imiquimod caused skin redness, thickness, and scaling. The control group showed no signs of psoriatic skin lesions, whereas the vehicle control group, which was subjected to imiquimod application but was untreated, presented the most serious psoriasis-like skin lesions. In the early treatment model, treatment groups with 5 mg/kg, 10 mg/kg, and 20 mg/kg of forsythoside A relieved the severity of psoriasis-like dorsal skin lesions, with decreased redness and scaling, similar to the group treated with dexamethasone (Figure 1A). Compared with the vehicle control group, the PASI scores of skin redness and scaling were all lower in the groups treated with different doses of forsythoside A. Ear thicknesses were also decreased more in the treatment groups compared with those in the vehicle control group (Figure 1B). In the treatment model on day 5, treatment group with 5 mg/kg of forsythoside A slightly relieved the severity of psoriasis-like dorsal skin lesions, whereas the groups with 10 mg/kg and 20 mg/kg of forsythoside A showed a more obvious relieved effect, similar to the group with dexamethasone. By day 8, the skin lesions seemed to be recovered in the vehicle control group, forsythoside A treatment groups, and dexamethasone group (Figure 1C). Compared with the vehicle control group, the PASI scores of skin redness and scaling were apparently lower in the groups treated with 10 mg/kg and 20 mg/kg of forsythoside A at day 5. Ear thicknesses were also decreased more in the treatment groups (Figure 1D). These results suggest that forsythoside A can effectively relieve psoriatic skin lesion symptoms such as skin redness, thickness, and scaling, both in the early treatment model and treatment model.

### 3.2. Forsythoside A Reduces Keratinocyte Proliferation and Pro-Inflammatory Cytokine Expression in Imiquimod-Induced Psoriasis-like Dermatitis

**Figure 2:**
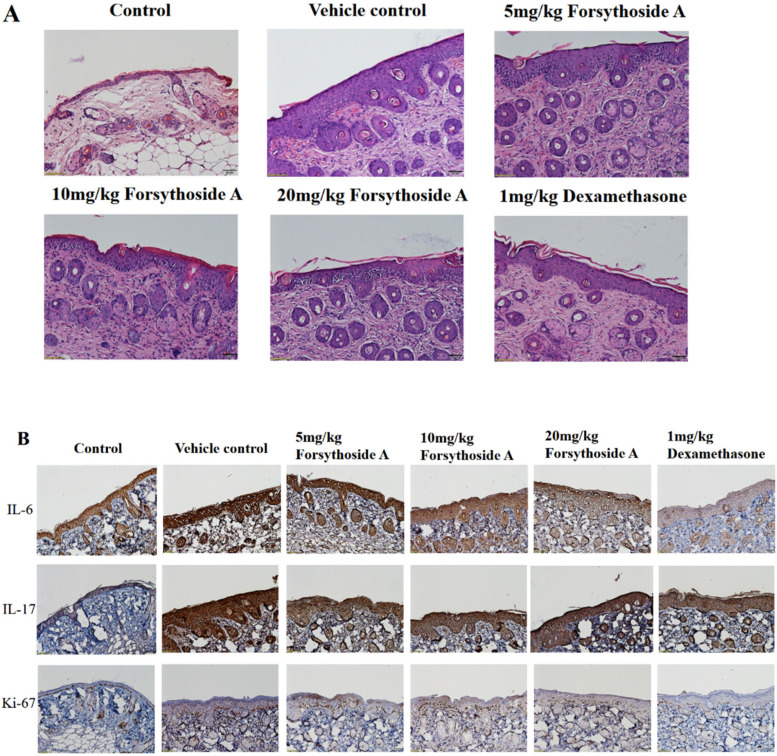

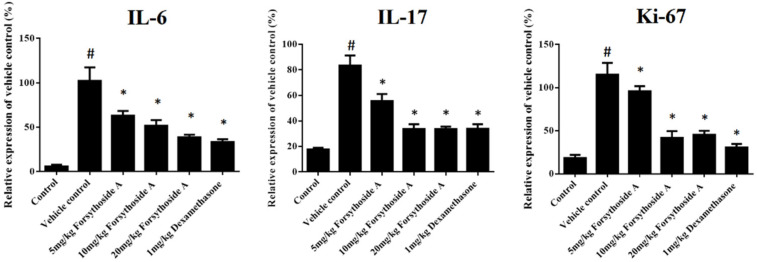
Forsythoside A improved the pathological features and reduced pro-inflammatory cytokine expression in the IMQ-induced psoriasis-like dermatitis lesions in mice. (**A**) Hematoxylin and eosin (H&E) staining of the dorsal skin (magnification 200×) and quantitation of epidermal thickness. (**B**) Immunohistochemistry (IHC) staining for IL-6, IL-17, and Ki-67 in the dorsal skin (magnification 200×). Mean optical densities of IL-6, IL-17 and Ki-67 are presented as the mean ± SEM (*n* = 5 in each group). # *p* < 0.05 vs. the control group; * *p* < 0.05 vs. the vehicle control group.

H&E staining was performed to observe the microenvironmental effects in skin lesions after treatment with forsythoside A. Imiquimod caused increased thickening of the epidermis layer and increased immune cell infiltration in the dermis layer, which was observed in the vehicle control group. Forsythoside A improved the pathological changes in skin lesions with reduced epidermal thickening, showing an effect similar to that in the dexamethasone group (Figure 2A). The IHC staining results showed increased expressions of IL-6, IL-17, and Ki-67 in the vehicle control group, which were all downregulated in the forsythoside A treatment groups (Figure 2B). These results suggest that forsythoside A can reduce keratinocyte proliferation and the expression of IL-6, IL-17, and Ki-67 in psoriasis-like dermatitis.

### 3.3. Forsythoside A Reduces Th17 Cells in Inguinal Lymph Nodes

**Figure 3:**
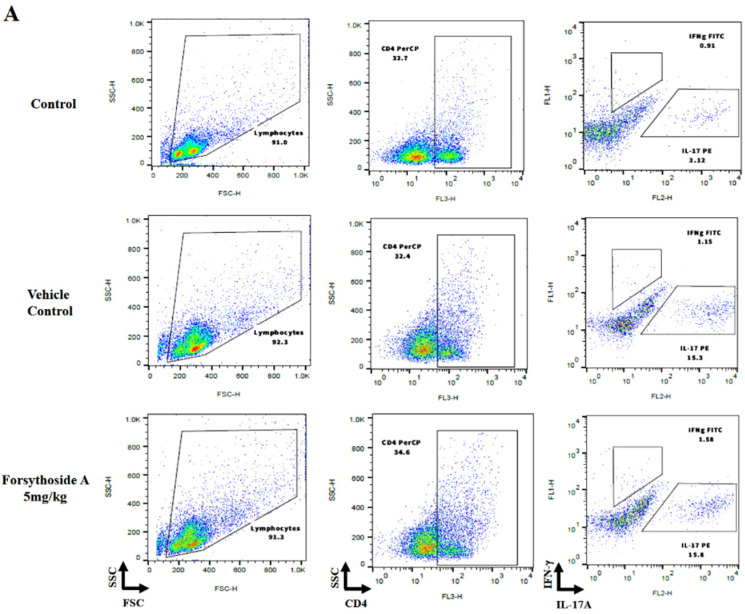

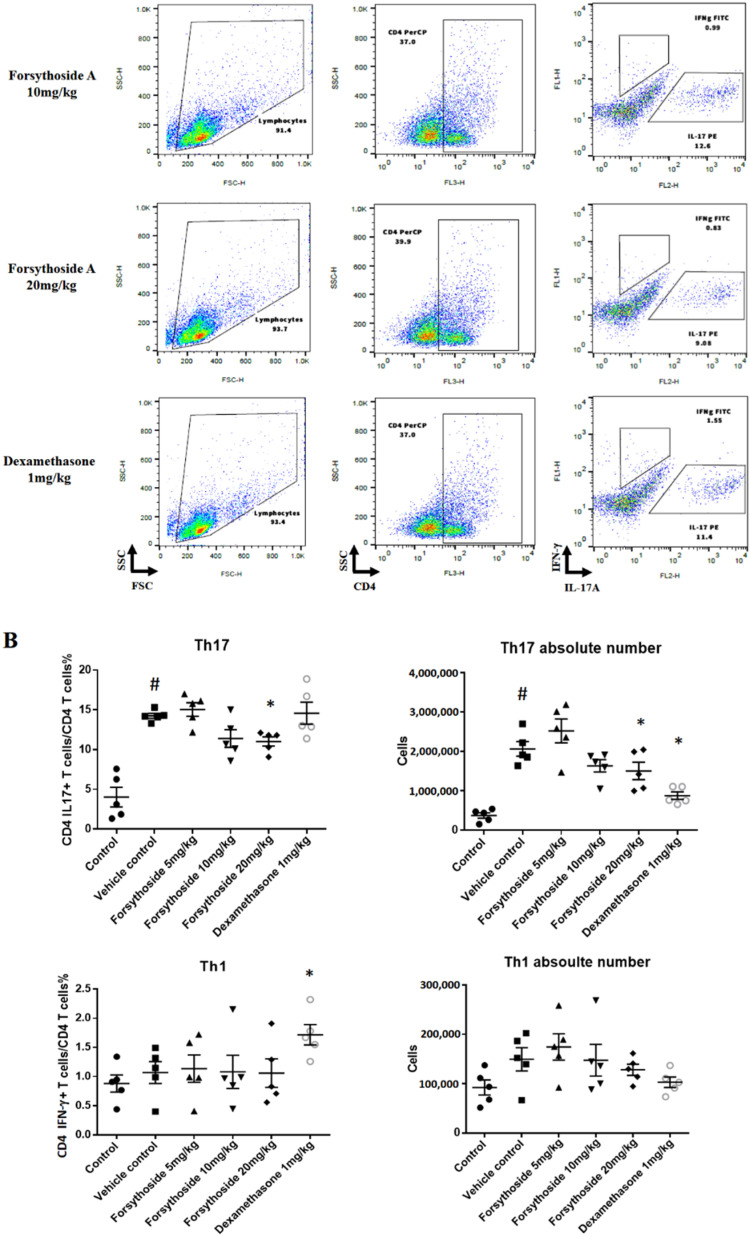
Forsythoside A reduced Th17 cells in inguinal lymph nodes. (**A**) Representative data and gating strategies for the flow cytometric analysis of CD4+ cells in inguinal lymph nodes. (**B**) Cytokine expression of CD4+ lymphocytes in inguinal lymph nodes were analyzed by flow cytometry and the absolute number of CD4+ lymphocytes was calculated. Data are presented as the mean ± SEM (*n* = 5 in each group). # *p* < 0.05 vs. the control group; * *p* < 0.05 vs. the vehicle control group.

Th1 and Th17 cell development is considered to play a key role in autoimmune diseases [12]. To explore the effect of forsythoside A on CD4+ T cells, we measured the percentage and absolute number of IFN-γ+ or IL-17+ in CD4+ T cells in inguinal lymph nodes by flow cytometric analysis. Representative images of the CD4+IFN-γ+ and CD4+IL-17+ percentages are shown in Figure 3A. The results showed that a high dose of forsythoside A (20 mg/kg) significantly reduced the percentage and absolute number of Th17 cells in inguinal lymph nodes, whereas the dexamethasone group only reduced the absolute number of Th17 cells. In contrast, forsythoside A did not significantly influence either the percentage or absolute number of Th1 cells (Figure 3B). These results suggest that forsythoside A suppresses Th17 cell in imiquimod-induced psoriasis-like dermatitis.

### 3.4. Forsythoside A Suppresses the Secretion of IL-17A In Vitro

**Figure 4:**
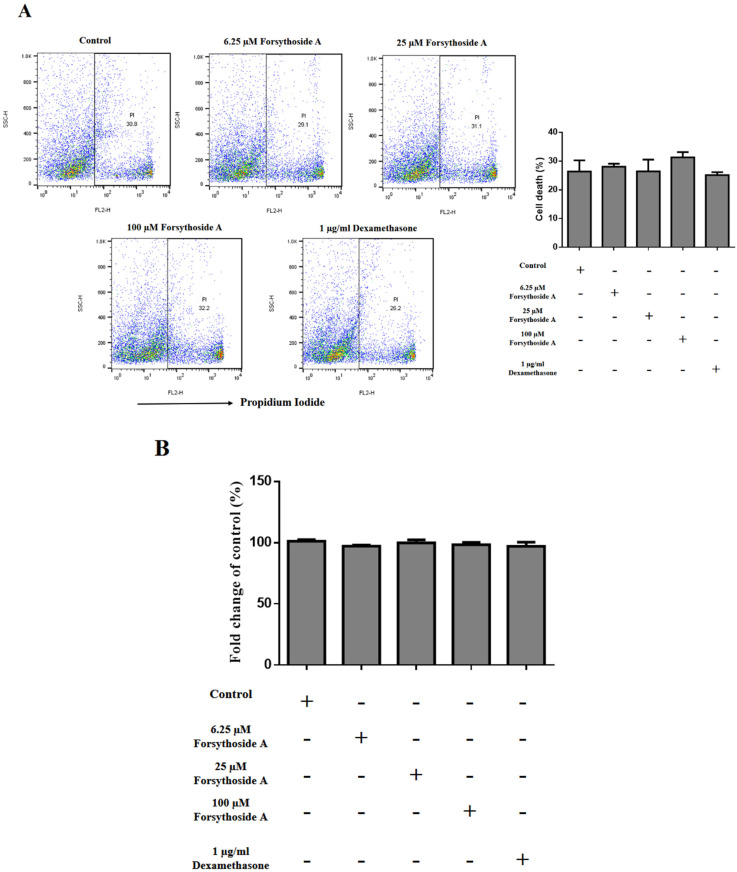

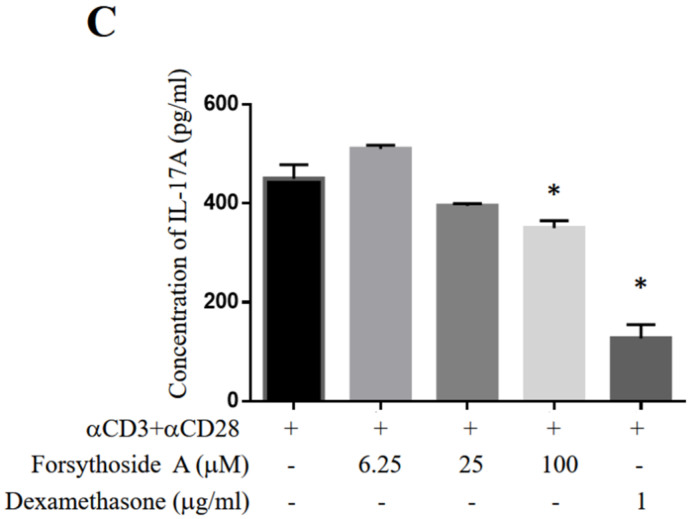
Cell viability and apoptosis of forsythoside-A-treated splenocytes was evaluated by (**A**) propidium iodide (PI) flow cytometric assay and (**B**) CCK-8 assay. (**C**) IL-17A secretion by forsythoside-A-treated lymphocytes. Lymphocytes isolated from inguinal lymph nodes were activated by plate-bound anti-mouse CD3 and CD28, and were then treated with different doses of forsythoside A for 24 h. The concentration of IL-17A in the supernatant was measured by ELISA. Data are presented as the mean ± SEM. * *p* < 0.05 vs. the control group. +: treated with indicated drugs or antibodies. -: none treated.

IL-17A plays a crucial role in psoriasis by stimulating the differentiation and proliferation of keratinocytes [13]. We first evaluated the cell viability and apoptosis of forsythoside-A-treated splenocytes by propidium iodide (PI) flow cytometric assay (Figure 4A) and CCK-8 assay (Figure 4B). The results showed that forsythoside A did not cause an adverse effect on cell survival compared with the control group. Subsequently, we examined the concentration of IL-17A in lymphocyte cultures to determine whether forsythoside A inhibits IL-17A secretion. The results showed that lymphocytes treated with 100 μM forsythoside A or 1 μg dexamethasone significantly lowered the concentration of IL-17A compared with the control (Figure 4C). These results suggest that forsythoside A can inhibit IL-17A secretion in vitro.

## 4. Discussion

Forsythoside A is a phenylethanoid glycoside isolated from the fruit of *Forsythia suspensa*. Previous studies have indicated its pharmacological effects, such as anti-inflammatory [10,14], antioxidant [15], and anti-endotoxin [16] activities. The application of forsythoside A to treat dermatitis was mentioned in previous study, by reducing the serum levels of histamine, TNF-α and IgE [10]. In the present study, we demonstrated the anti-psoriatic effects of forsythoside A in vivo and in vitro. Forsythoside A significantly reduced the thickness of the epidermal layer and improved clinical psoriatic skin symptoms such as skin redness, thickness, and scaling. Furthermore, forsythoside A may affect CD4+ T cell differentiation by suppressing Th17 cell expression and reducing IL-17A secretion. Previous studies indicated the effect of forsythoside A to suppress T cells from differentiating into Th17 cells in influenza-A-infected mice [11,17].

The dose of forsythoside A applied in our study ranged from 5 mg/kg to 20 mg/kg, considering that a dose exceeding 25 mg/kg may cause pseudoallergic reactions [18]. In the treatment model, one mouse in the treatment group with 5 mg/kg forsythoside A died at day 5 for unknown reasons. The PASI scores and ear thicknesses of four other mice were still recorded from day 6 to day 8.

Imiquimod-induced psoriasis-like dermatitis in mice is a widely used model for studying human psoriasis [19,20], because it mimics the immune imbalance of psoriasis. Among its complex pathogenetic mechanisms, psoriasis is mainly believed to be a Th17-cell-mediated inflammatory disease. A previous study indicated that the inflammatory environment of psoriasis mainly contains Th1 and Th17 cells [21], and that Th17 cells may play a key role. Th17 cells are differentiated in psoriatic skin lesions and are activated by the polarizing effects of IL-6, IL-23, IL-1, and other cytokines produced by inflammatory dendritic cells, leading to the secretion of proinflammatory cytokines such as IL-17A, IL-22, and IL-26, which induce keratinocyte proliferation [22], contributing to the development of the psoriatic skin phenotype. As indicated by flow cytometric analysis and CCK-8 assay, we discovered that forsythoside A significantly suppressed Th17 cell development in imiquimod-induced psoriasis mice, both in percentages and absolute numbers, causing no adverse effect on cell survival compared with the control group. This may indicate that forsythoside A is capable of treating psoriasis by regulating Th17 cell development and activation, although further investigation of the definite mechanism is needed.

IL-6 plays a crucial role in determining the balance between IL-17-producing Th17 cells and regulatory T cells [23]. A previous study claimed that IL-6 trans-presentation by dendritic cells is required to produce pathogenic Th17 cells [24]. Another study reported high levels of IL-6 secreted spontaneously by dermal cells from psoriatic skin lesions compared with those from non-lesional skin, and found abundant IL-17-producing cells co-expressing IL-6 [25]. Therefore, targeting IL-6 and IL-17 may be a promising strategy for the treatment of psoriasis. The present study demonstrates the downregulating effects of forsythoside A on IL-6 and IL-17 expression, indicating that forsythoside A can ease psoriasis-like dermatitis by suppressing the secretion or expression of IL-6 and IL-17.

Psoriasis is an autoimmune skin disease characterized by recurrent epidermal hyperplasia. Ki-67 is a widely used proliferation marker for human tumor cells [26]. Another study showed that Ki-67 is a feasible marker of proliferation in the initial phase of adult neurogenesis [27]. Furthermore, previous studies have claimed that reduced Ki-67 expression was discovered after different kinds of treatment were applied to psoriatic skin lesions [28,29]. In this study, the results showed reduced Ki-67 expression after treatment with forsythoside A, implying that it inhibits epidermal proliferation in psoriatic skin lesions.

IL-17A is a cytokine secreted by Th17 cells and is abundantly found in the lesions of patients with psoriasis. A previous study indicated the crucial role of IL-17A in the pathogenesis of human psoriasis [30]. It can cause the abnormal proliferation and differentiation of keratinocytes, later contributing to a positive feedback cycle of inflammatory status in psoriasis [31]. Hence, the development of anti-IL-17A drugs has become a promising strategy for psoriatic treatment. Secukinumab and Ixekizumab are two biological agents clinically available for psoriasis, which can relieve inflammation by selectively binding to IL-17A. In the present study, we found that 100 μM of forsythoside A could reduce the IL-17A concentration in vitro, suggesting the possibility of forsythoside A as a new promising small-molecule drug for psoriasis that targets IL-17A.

This study demonstrated that forsythoside A significantly relieved psoriasis-like dermatitis in imiquimod-induced murine models. However, there are some limitations to our study. First, the inflammatory status of psoriasis-like skin lesions, including skin redness and scaling, peaked before the day of sacrifice. Earlier sacrifice should thus be considered to explore more significant regulatory effects of the immune system. Second, the skin of the control group looked damaged, which may have been caused by hair removal. The hair removal method should be improved in further experiments. Third, clinical trials examining the effect of forsythoside A on patients with psoriasis are necessary to study its effects and safety in humans. Finally, the mechanism by which forsythoside A suppresses Th17 cell development and IL-17A expression remains unclear. Further experiments are thus required to clarify the mechanisms underlying the effect of forsythoside A in psoriasis treatment.

## 5. Conclusions

In conclusion, our study demonstrates that forsythoside A alleviates imiquimod-induced psoriasis-like dermatitis, mainly by suppressing Th17 development and the expression of pro-inflammatory cytokines. These findings demonstrate the feasibility of forsythoside A for human psoriasis treatment and its potential therapeutic effects against other inflammatory diseases related to Th17 and IL-17A.

## Data Availability

The datasets used and/or analyzed during the present study are available from the corresponding author upon reasonable request.

## References

[1] Parisi R., Symmons D.P., Griffiths C.E., Ashcroft D.M. (2013). Global epidemiology of psoriasis: A systematic review of incidence and prevalence. J. Investig. Dermatol..

[2] Michalek I.M., Loring B., John S.M. (2017). A systematic review of worldwide epidemiology of psoriasis. J. Eur. Acad. Dermatol. Venereol. JEADV.

[3] Langley R.G., Krueger G.G., Griffiths C.E. (2005). Psoriasis: Epidemiology, clinical features, and quality of life. Ann. Rheum. Dis..

[4] Rendon A., Schäkel K. (2019). Psoriasis pathogenesis and treatment. Int. J. Mol. Sci..

[5] Takeshita J., Grewal S., Langan S.M., Mehta N.N., Ogdie A., Van Voorhees A.S., Gelfand J.M. (2017). Psoriasis and comorbid diseases: Epidemiology. J. Am. Acad. Dermatol..

[6] Hawkes J.E., Chan T.C., Krueger J.G. (2017). Psoriasis pathogenesis and the development of novel targeted immune therapies. J. Allergy Clin. Immunol..

[7] Hawkes J.E., Yan B.Y., Chan T.C., Krueger J.G. (2018). Discovery of the il-23/il-17 signaling pathway and the treatment of psoriasis. J. Immunol..

[8] Wollina U., Tirant M., Vojvodic A., Lotti T. (2019). Treatment of psoriasis: Novel approaches to topical delivery. Open Access Maced. J. Med. Sci..

[9] Gisondi P., Del Giglio M., Girolomoni G. (2017). Treatment approaches to moderate to severe psoriasis. Int. J. Mol. Sci..

[10] Sung Y.Y., Yoon T., Jang S., Kim H.K. (2016). *Forsythia suspensa* suppresses house dust mite extract-induced atopic dermatitis in nc/nga mice. PLoS ONE.

[11] Deng L., Pang P., Zheng K., Nie J., Xu H., Wu S., Chen J., Chen X. (2016). Forsythoside a controls influenza a virus infection and improves the prognosis by inhibiting virus replication in mice. Molecules.

[12] Leung S., Liu X., Fang L., Chen X., Guo T., Zhang J. (2010). The cytokine milieu in the interplay of pathogenic th1/th17 cells and regulatory t cells in autoimmune disease. Cell. Mol. Immunol..

[13] Furue M., Furue K., Tsuji G., Nakahara T. (2020). Interleukin-17a and keratinocytes in psoriasis. Int. J. Mol. Sci..

[14] Zhang X.T., Ding Y., Kang P., Zhang X.Y., Zhang T. (2018). Forsythoside a modulates zymosan-induced peritonitis in mice. Molecules.

[15] Guo Y.P., Lin L.G., Wang Y.T. (2015). Chemistry and pharmacology of the herb pair *Flos Lonicerae japonicae*-*Forsythiae fructus*. Chin. Med..

[16] Zeng X.Y., Yuan W., Zhou L., Wang S.X., Xie Y., Fu Y.J. (2017). Forsythoside a exerts an anti-endotoxin effect by blocking the lps/tlr4 signaling pathway and inhibiting tregs in vitro. Int. J. Mol. Med..

[17] Zheng X., Fu Y., Shi S.S., Wu S., Yan Y., Xu L., Wang Y., Jiang Z. (2019). Effect of forsythiaside a on the rlrs signaling pathway in the lungs of mice infected with the influenza a virus fm1 strain. Molecules.

[18] Han J., Zhang Y., Pan C., Xian Z., Pan C., Zhao Y., Li C., Yi Y., Wang L., Tian J. (2019). Forsythoside a and forsythoside b contribute to shuanghuanglian injection-induced pseudoallergic reactions through the rhoa/rock signaling pathway. Int. J. Mol. Sci..

[19] Yang B.Y., Cheng Y.G., Liu Y., Liu Y., Tan J.Y., Guan W., Guo S., Kuang H.X. (2019). *Datura Metel* L. Ameliorates imiquimod-induced psoriasis-like dermatitis and inhibits inflammatory cytokines production through tlr7/8-myd88-nf-κb-nlrp3 inflammasome pathway. Molecules.

[20] Wu S., Zhao M., Sun Y., Xie M., Le K., Xu M., Huang C. (2020). The potential of diosgenin in treating psoriasis: Studies from hacat keratinocytes and imiquimod-induced murine model. Life Sci..

[21] Lowes M.A., Kikuchi T., Fuentes-Duculan J., Cardinale I., Zaba L.C., Haider A.S., Bowman E.P., Krueger J.G. (2008). Psoriasis vulgaris lesions contain discrete populations of th1 and th17 t cells. J. Investig. Dermatol..

[22] Di Cesare A., Di Meglio P., Nestle F.O. (2009). The il-23/th17 axis in the immunopathogenesis of psoriasis. J. Investig. Dermatol..

[23] Kimura A., Kishimoto T. (2010). Il-6: Regulator of treg/th17 balance. Eur. J. Immunol..

[24] Heink S., Yogev N., Garbers C., Herwerth M., Aly L., Gasperi C., Husterer V., Croxford A.L., Möller-Hackbarth K., Bartsch H.S. (2017). Trans-presentation of il-6 by dendritic cells is required for the priming of pathogenic t(h)17 cells. Nat. Immunol..

[25] Goodman W.A., Levine A.D., Massari J.V., Sugiyama H., McCormick T.S., Cooper K.D. (2009). Il-6 signaling in psoriasis prevents immune suppression by regulatory t cells. J. Immunol..

[26] Sun X., Kaufman P.D. (2018). Ki-67: More than a proliferation marker. Chromosoma.

[27] Kee N., Sivalingam S., Boonstra R., Wojtowicz J.M. (2002). The utility of ki-67 and brdu as proliferative markers of adult neurogenesis. J. Neurosci. Methods.

[28] Jesionek-Kupnicka D., Chomiczewska-Skóra D., Rotsztejn H. (2013). Influence of phototherapy in psoriasis on ki-67 antigen expression: A preliminary study. Pol. J. Pathol. Off. J. Pol. Soc. Pathol..

[29] Jang H.S., Oh C.K., Jo J.H., Kim Y.S., Kwon K.S. (2001). Detection of telomerase activity in psoriasis lesional skin and correlation with ki-67 expression and suppression by retinoic acid. J. Korean Med. Sci..

[30] Brembilla N.C., Senra L., Boehncke W.H. (2018). The il-17 family of cytokines in psoriasis: Il-17a and beyond. Front. Immunol..

[31] Martin D.A., Towne J.E., Kricorian G., Klekotka P., Gudjonsson J.E., Krueger J.G., Russell C.B. (2013). The emerging role of il-17 in the pathogenesis of psoriasis: Preclinical and clinical findings. J. Investig. Dermatol..

